# P07 Study of Prescribing patterns and Effectiveness of Ceftolozane/Tazobactam Real-world Analysis (SPECTRA): results on critical care patients from a multinational, multicentre, observational analysis

**DOI:** 10.1093/jacamr/dlae136.011

**Published:** 2024-08-23

**Authors:** Alejandro Soriano, David L Paterson, Florian Thalhalmmer, Stefan Kluge, Pierluigi Viale, Brune Akrich, Mike Allen, Stephanie Wirbel, Engels N Obi, Sunny Kaul

**Affiliations:** Department of Infectious Diseases, Hospital Clinic, Barcelona, Spain; Centre for Clinical Research, The University of Queensland, Queensland, Australia; Department of Urology, Medical University of Vienna, Wien, Austria; Department of Intensive Care Medicine, University Medical Center Hamburg-Eppendorf, Hamburg, Germany; Department of Medical and Surgical Sciences, University of Bologna, Bologna, Italy; Medical Affairs, MSD, Puteaux, France; Medical Affairs, MSD, London, UK; Biostatistics & Medical Writing, ICON Commercialization & Outcomes, ICON Plc, Lyon, France; Center for Observational and Real-world Evidence, Merck & Co. Inc., Rahway NJ, USA; Dept of Respiratory Medicine and Intensive Care, Harefield Hospital, London, UK

## Abstract

**Background:**

Ceftolozane/tazobactam (C/T) is indicated for the treatment of complicated intra-abdominal infections (cIAI),^1^ complicated urinary tract infections (cUTI), including pyelonephritis,^2^ and hospital-acquired bacterial and ventilator-associated bacterial pneumonia.^3^ Information on real-world use and outcomes of patients treated with C/T in critical care settings is important to help inform disease management and clinical practice.

**Objectives:**

This study presents findings on patient/treatment characteristics and outcomes associated with C/T use in patients managed in the critical care setting.

**Methods:**

Data were collected from the SPECTRA study, a multinational, multicentre, retrospective, inpatient, observational study of patients treated with C/T across hospitals in seven countries (Spain, the UK, Germany, Italy, Austria, Australia and Mexico) from 2016–2020. All adult patients admitted to the ICU during the index hospitalization and treated with C/T for ≥48 h were included. Demographics, clinical characteristics, treatment management patterns and outcomes were assessed.

**Results:**

The study sample included 298 critical care patients receiving C/T (mean age 57.0 years; 68.8% male), 41.9% of which had an infection-related ICU admission. Sites of infection were respiratory (50.0%), skin/wound/tissue (21.1%), blood (13.7%), urine (10.3%), pleural fluid/cerebrospinal fluid/other fluid (9.3%), line/device (2.9%), abdominal (1.0%), bone/joint (0.5%) and stool (0.5%). The most common pathogens were *Pseudomonas aeruginosa* (89.7%) and *Escherichia coli* (6.4%). Most patients (81.5%) had at least one comorbidity, with the most common being immunocompromised state (44.6%), sepsis (41.6%), heart disease (29.2%) and chronic pulmonary disease (27.2%). Renal replacement therapy was initiated in 21.5% of patients during index hospitalization, with 14.4% on continuous renal replacement therapy. The most common C/T regimen was 1.5 g every 8 h (36.9% of patients). About 51.6% of patients received C/T as third line or salvage (24.8% and 23.5% received C/T as first or second line, respectively). Median C/T treatment duration was 11.0 days (Q1, Q3: 7.0, 16.5 days). Clinical success was 53.4%. All-cause in-hospital mortality was 35.6% overall, and 13.8% infection related. Thirty-day all-cause readmission was 3.4% overall, and 1.7% infection related.

**Conclusions:**

This multinational real-world study included a high number of critical care patients and reported outcomes associated with C/T use. In this very high-risk cohort presenting with severe Gram-negative infections, most C/T patients had beneficial outcomes despite their clinical complexity and late intervention with C/T.

## References

[1] Solomkin J et al Ceftolozane/tazobactam plus metronidazole for complicated intra-abdominal infections in an era of multidrug resistance: results from a randomized, double-blind, Phase 3 trial (ASPECT-cIAI). Clin Infect Dis 2015; 60: 1462–71. 25670823 10.1093/cid/civ097 PMC4412191

[2] Wagenlehner FM et al Ceftolozane-tazobactam compared with levofloxacin in the treatment of complicated urinary-tract infections, including pyelonephritis: a randomised, double-blind, Phase 3 trial (ASPECT-cUTI). Lancet 2015; 385: 1949–56. 25931244 10.1016/S0140-6736(14)62220-0

[3] Kollef MH et al Ceftolozane-tazobactam versus meropenem for treatment of nosocomial pneumonia (ASPECT-NP): a randomised, controlled, double-blind, Phase 3, non-inferiority trial. Lancet Infect Dis 2019; 19: 1299–1311. 31563344 10.1016/S1473-3099(19)30403-7

